# Burnout and the role of authentic leadership in academic medicine

**DOI:** 10.1186/s12913-022-08034-x

**Published:** 2022-05-11

**Authors:** Katie McPherson, Juliana G. Barnard, Martha Tenney, Brooke Dorsey Holliman, Katherine Morrison, Patrick Kneeland, Chen-Tan Lin, Marc Moss

**Affiliations:** 1 Department of Medicine, Vanderbilt University, Nashville, TN USA; 2 Adult and Child Center for Outcomes Research and Delivery Science (ACCORDS), University of Colorado, Aurora, CO USA; 3 Department of Pediatrics, University of Colorado School of Medicine, Aurora, CO USA; 4 Mined Insights Consulting LLC, Denver, CO USA; 5 Department of Medicine, University of Colorado, Aurora, CO USA; 6 Department of Family Medicine, University of Colorado School of Medicine, Aurora, CO USA; 7 Division of Hospital Medicine, University of Colorado School of Medicine, Aurora, CO USA; 8 Dispatch Health, Denver, CO USA; 9 Division of Pulmonary Sciences and Critical Care Medicine, Research 2, Box C272, 9th floor, 12700 E. 19th Avenue, Aurora, CO 80045 USA

**Keywords:** Authentic leadership, Healthcare workers burnout

## Abstract

**Background:**

Recently, there has been increasing evidence that reducing burnout in healthcare providers requires significant organizational efforts that include the integration of leadership strategies.

**Methods:**

Focus groups were conducted across four health systems within the University of Colorado Department of Medicine in four affinity groups (administrative staff, medical trainees, research faculty, and clinical faculty). Authentic leadership theory was used for analysis to advance the understanding of the role of leadership style upon participants’ work experiences and preferences, and to identify opportunities for translation of site-specific results to other academic medical settings.

**Results:**

Study participants from each affinity group believed their clinical leaders lacked objectivity with decision-making (lacking “balancing processing”), which contributed to their overall feeling of powerlessness. The experience of increasing work demands was salient throughout all twelve focus groups, and participants identified leadership that interacted in a more open and self-disclosing manner (“relational transparency”) as alleviating at least some of this burden. Strong preference discernable alignment between their leaders’ decision-making and their internal moral compass of values (demonstrating “internalized moral perspective”) was described, as was clinical leaders demonstrating “self-awareness” (having a self-reflective process that informs the leader’s decision-making). Comparing affinity group experiences within each authentic leadership theory construct identified the relevance of contextual factors, such as work setting and roles, upon employees’ perceptions and expectations of their leaders.

**Conclusions:**

Use of authentic leadership theory advanced the understanding of the association between leadership traits and experiences of burnout amongst a large group of academic clinicians, researchers, trainees, and administrative staff. Leadership styles that promoted relationship transparency, openness, and support were preferred and fostering these traits may help address the demands in academic medicine, including symptoms of burnout.

**Supplementary Information:**

The online version contains supplementary material available at 10.1186/s12913-022-08034-x.

Burnout syndrome is a deleterious work-related mental health condition that occurs among individuals without any prior history of psychological disorders [1]. As stress in the healthcare setting has increased dramatically, burnout is reaching epidemic proportions in healthcare professionals [2–4]. The etiology of burnout is complex, though symptoms of burnout are related to organizational factors including amount of workload, autonomy and control over practice, quality of the work environment, and shared governance [5]. Healthcare professionals in academic settings face particular challenges related to burnout, given the multifaceted and sometimes conflicting missions of patient care, research, and education. In addition, the individuals who create and contribute to this institutional mission are diverse and have unique needs that must be met to thrive in their professional lives. These individuals include trainees, clinical faculty, researchers, and administrative staff.

Though many conceptual models exist for burnout and work-related well-being, the National Academy of Medicine (NAM) developed their own model that applies across all healthcare professionals and career stages [5]. Their model focused on the relationship between well-being and outcomes for clinicians, patients, and healthcare systems [5–9]. The NAM model includes external factors (socio-cultural; regulatory, business, and payer environment; organizational; and learning/practice environment), and individual factors (healthcare role, personal factors, and skills and abilities) [5]. One of the overarching themes that resonated across several of the model domains is leadership. Effective leadership styles can likely mitigate many triggers of burnout by enhancing autonomy and control, empowering individuals, and optimizing the work environment [10–13]. There is a relative paucity of data about how specific leadership styles impact burnout in healthcare workers, particularly among leaders who are pursuing a multidimensional academic mission [14, 15]. A review of the leadership theory literature suggests that authentic leadership may be an ideal style to mitigate burnout [16–19]. Authentic leadership is defined as a process that draws from both positive psychological capacities and a highly developed organizational context, which results in both greater self-awareness and self-regulated positive behaviors on the part of leaders and associates, thereby fostering positive self-development [20]. Authentic leadership influences staff attitudes and behaviors through the key psychological processes of identification, hope, positive emotions, optimism, and trust [16].

To our knowledge, few studies have explored the possible implications of leadership style on burnout through the lens of a variety of members of an academic department of medicine [21, 22]. With this research question in mind, we conducted a qualitative study following content analysis as the methodological orientation. Focus groups methods were used across four health systems of the University of Colorado School of Medicine Department of Medicine (DOM) to identify leadership attributes that were postulated to be associated with a reduction in burnout syndrome and methods to foster feeling more connected to the academic mission. The authentic leadership theory was used to analyze our findings by its constructs and to identify opportunities for the translation of site-specific results to other academic medical settings.

## Methods

We followed the COREQ (COnsolidated criteria for REporting Qualitative research) Checklist for reporting our study methods [23]. Twelve focus groups were conducted from November 2018 to January 2019. Participants were recruited by multiple methods, including email, departmental newsletter, and in-person recruitment performed by DOM wellness group members. Focus groups were conducted on-site at four health systems where DOM members have appointments including University of Colorado Hospital (UCHealth), Rocky Mountain VA Medical Center, National Jewish Health, and Denver Health in four affinity clusters: trainees (residents and fellows), clinical faculty, researchers (including research nurses), and administrative staff (business/grants staff and research assistants). Focus group were conducted by a focus group facilitator (MT) and a note taker (KMcP), neither had any prior work relationship with the study participants.

The focus group guide was developed through both a review of medical literature and by a multidisciplinary team (authors KMcP, KM, CTL, PK, a qualitative research consultant MT, and members of DOM Wellness committee) for all affinity clusters. It was piloted and revisions incorporated before use. Questions focused on current experiences with a culture of wellness, methods of connection, and leadership qualities (See Attachment). Recordings were transcribed, checked for accuracy, and analyzed in ATLAS.ti© v8 qualitative data software.

### Analytic methods

Analysis of the qualitative data was team-based, used both inductive and deductive approaches, and continued until thematic saturation was reached during two phases. The research team initially coded de-identified transcripts, discussed coded data, and reviewed similarities and differences until consensus was reached. Subsequent axial coding and ongoing discussions allowed for emergence of contextual patterns and themes. We also reviewed emerging themes and included exploration of confirming and disconfirming data.

Findings from the first round of analysis were re-analyzed using authentic leadership theory constructs to deepen the understanding of data from a systems-level theory perspective. Use of an analytic theory facilitated the authors’ understanding of how the study findings were and were not related to results of other studies, and other clinical settings. The results were analyzed and are presented using the authentic leadership constructs, and by comparing results by affinity groups for each theoretical construct.

## Results

Seventy-one DOM employees participated in 12 focus groups (See Table 1). Findings supported the constructs of authentic leadership theory (“balanced processing”, “relational transparency”, “internalized moral perspective”, and “self-awareness”) and were related to a varying range of work attitudes (eg, job satisfaction, work engagement, well-being) and, to a lesser extent, behaviors (eg, extra-role behavior, knowledge sharing, changing jobs).

**Table 1:** Characteristics of study participants and focus groups

***Affinity Group***	***# of Focus Groups (FG)***	***# of FG Participants (range/FG)***	***# FGs by Location***
**Clinical Faculty**	4 focus groups	*N* = 22	2-Anschutz
		Range: 4–7	1-National Jewish Health
			1-Denver Health MC
**Trainees**	3 focus groups	*N* = 18	3-Anschutz
		Range: 4–10	
**Staff**	3 focus groups	*N* = 20	3-Anschutz
		Range: 5–8	
**Research Faculty**	2 focus groups	*N* = 11	1-Anschutz
		Range: 5–6	1-National Jewish Health
**Total**	12 focus groups	*N* = 71	9-Anschutz
		Range: 4–10	2-National Jewish Health
			1-Denver Health MC

### Balanced processing

The balanced processing construct emphasizes the importance of objective leadership [13, 14]. A defining characteristic of this leadership style is consideration of all relevant information before making decisions. Leaders with this trait are open to soliciting views that may challenge their own position. Study participants from each affinity group described circumstances in which leaders demonstrated balanced processing and were disappointed when their leaders’ lacked consideration for participants opinions. Participants reported that they tended to feel powerless when their leaders lacked balanced processing leadership traits.

Clinical faculty participants described during training when their input was considered and used to implement change. These experiences with leadership were preferred; they set the tone for shared decision-making and a sense of agency. For instance, one clinical faculty member said that “having leaders who actually enact changes in the division or the work environment, concrete changes, based on feedback that we provide” is important to determine their interest in feedback. In contrast, the more common and current leadership style they encountered was a top-down approach of enforcing policy change without group input or understanding of the workplace, leading to what participants described as poor adoption. When leadership lacked knowledge of the day-to-day realities of clinical work, it prevented them from using relevant information important to understanding local context for their employees. This was highlighted by a clinical faculty member who said, “it feels like we’re quite removed from the …hospital leadership that it – like at a bigger level--our day-to-day is here…I think that can contribute to feelings of being misunderstood.” The clinical faculty and trainees agreed that an increase in the approachability of the leadership would improve their overall wellness and positively impact patient care.

Among trainees, the characteristics of various departmental meetings were discussed. For example, some divisions conduct meetings to gain input and consensus from division members about specific topics or operational changes. A trainee pointed out that after these meetings, “We actually see changes within the next couple of months to academic year. And I think that’s really, really important to feel like, one, you have access to the leadership, and two, not only are you expressing your opinions (which is easy to do) but then that they’re doing something about it.”

These trainees agreed that having leaders who are willing to be a sounding board is nice but felt that leadership who took action and worked towards implementing changes they recommended helped them feel valued at work.

Yet most often, trainees described a lack of balanced processing leadership, which not only led to frustration, but also feelings of powerlessness (see Table 2 for supplementary illustrative quotations for all constructs). Study participants described feeling powerless because they believed their leadership thinks trainees are privileged to be in their positions and as a result, do not need to respond to their suggestions. Their leadership does not “think they’re going to lose quality--and they won’t--because that’s not something we (trainees) are willing to compromise”, even though leadership does not respond to their feedback, one trainee explained.

**Table 2:** Supplementary illustrative quotations

Construct of Authentic Leadership Theory	Quotation	Participant Type
*Balanced Processing:* Leadership’s lack of engaging employees in decision-making makes them feel powerless	*Participant A*: “I feel like they know that we have to be there, so they don’t really invest much in keeping us happy and pleased at work because we can’t really do anything about it. That’s how I feel--kind of disenfranchised. I don’t really have much agency in actually making any change. You’re just kind of this cog, but you can’t really do anything about it because you don’t have the power.” *Participant B*: “We just have no negotiating power, if you will. And so to the hospital, it feels like we’re just cheap labor to get the work done…like we’re in this training program until we’re done with it. And if we don’t finish it, then we’re not a trained physician; we can’t go get an actual job. We have no power (to influence decisions).”	Trainees
*Relational Transparency:* Honesty from Leaders about faculty ideas	*Participant A: “*If the first issue that you raised were better addressed and there were better communication and transparency and inclusiveness [from the clinical leadership], you wouldn’t need—so, the thing is that you can get the unintended consequence of becoming sort of a lobby group with an antagonistic thing going on.” *Participant B:* “…if they [the leadership] had the transparency and they were open to us and kept us informed, we wouldn’t need another [advocacy] group. This would be [needed] if they’re not willing to do that.”	Clinical Faculty
*Internalized Moral Perspective:* Leadership values and shows trust in staff	It’s not enough just to pay lip service [to the importance of clinical care]. You have to have something to back that up.	Clinical Faculty
*Self-Awareness:* Leadership encourages work-life balance	One faculty member explained a previous experience this way: “…when you signed your letter of offer, it said ‘60 hours’ and then the Dean would lean in and say ‘Of course, if you’re going to be successful and advance, you’re going to work 70 or 80 hours a week.’ And so, when I came here [and no one in Leadership expected 70–80 hours/week], I realized that people, in part, take—they are better about putting some limits around their practice so that they have that personal time.”	Clinical Faculty

### Affinity group comparison-balanced processing

Comparing balanced processing amongst the affinity groups suggests differences between the groups. Each group shared compelling examples of dissatisfaction with leadership’s decision making when it lacked balanced processing. The difference between groups was how balanced processing was described in ways relevant for the affinity groups’ work setting and context. Trainees described wanting the leadership to respond to their requests before the end of the academic year when voiced during their training rotations. The clinical and research faculty wanted leadership to consult them and incorporate their feedback about making changes relevant to their careers (such as promotion processes and requirements) and work setting. Differences in experiences relative to balanced processing were described by staff. Since staff tended to not have direct lines of communication with upper-level leadership, they did not have the opportunity to share relevant information about their workplace setting (and thus, no expectation that their leadership solicits and responds to their suggestions as described by balanced processing). As a result, they described creating workarounds to meet the needs of their work setting (education of residents and fellows). Internal accounting tracked fellows’ and residents’ hours only when in hospital, yet “since we are an academic institute, we try to educate the Fellow…It doesn’t matter if you’re in the hospital here or [affiliate location] or VA…To get support we always have to be very creative [in counting hours present]”. The staffs’ leaders exhibited an absence of the defining feature of balanced processing; they did not consider all relevant information before making decisions about tracking trainees’ clinical hours.

### Relational transparency

The relational transparency construct within authentic leadership theory describes the benefits of leaders interacting with their employees in ways that demonstrate a high degree of openness, and exhibit self-disclosure [16, 24]. These leadership traits have the effect of engendering a sense of trust amongst the work group. The participants in this study described selected and persuasive experiences during which their leaders exhibited relational transparency. Yet, these behaviors were described mostly as aspirational; meaning—leadership traits they reported would be ideal yet were rarely experienced, in their unit or research setting.

Valuing relational transparency—and recognizing its absence in current leaders—was described by members of each affinity group in our study. Experiences of openness and self-disclosure from leaders were described as instrumental to trainees to develop their own approach to challenging situations. It was particularly reassuring to participants when a supervisor interacted with them by simultaneously recognizing a difficult situation and acknowledging that they too had experienced the same challenges. Additionally, when told by their leaders that their emotions were understandable and acceptable, participants described it as important for their ability to remain healthy within demanding work settings. For example, a clinical faculty member described a patient’s death that was particularly hard to handle when she/he was a fellow: “When she died my attending just—she cried. And then that gave me the permission that… it was okay to feel sad and take the time to experience that…I think just the fact that we are not devoid of our personhood…I’ve found that to be one of the most helpful things.”

When present, experiences of leaders who were self-disclosing helped these employees embrace their own experiences and trust in their own abilities to handle work demands. Participants described a “transparent” leader as someone who does “not keep us in the dark about current events that can help us understand about increased demands on us.” Having a leader be transparent about constraints and pressure was valued by each affinity group. Having increased demands at work was described prevalently throughout all twelve focus groups. Failure by leaders to demonstrate relational transparency appeared to have negatively impacted the quality of interaction between employees, their sense of wellness, and tended to promote more adversarial interactions amongst employees (see Table 2).

### Affinity group comparison-relational transparency

As noted above, participants discussed aspirational but rare experiences with their leaders demonstrating relational transparency leadership traits. No differences between the affinity groups were expressed by these participants.

### Internalized moral perspective

The construct of a leader’s internalized moral perspective describes leadership that is based not in the organization’s directives or external rewards for its leaders, but rather in the leader’s internal moral compass that employees see as guiding their decision-making [25]. The consistency between a leader’s stated beliefs and actions are used by employees to judge a leader’s genuineness, and are an expression of the leader’s internalized moral character or perspective.

Across all affinity groups, participants described preference for their leaders to make decisions that followed their internal morality. If wellness is a part of what a leader says they care about, then to have consistency between those beliefs and actions was important to these participants. As highlighted by a research faculty member, “actually demonstrating that it’s important by asking about it [wellness] and also helping to foster it in the people one supervises” was meaningful. Further, when leaders demonstrated an internalized moral perspective, participants told us it engendered a level of trust and encouraged employees’ support. As one trainee said, “I think that people talk a lot about wellness and taking time off to do things that you need. But then actually seeing one of your leaders do it is actually [encouraging], like ‘Okay, well, we should all do this, too.’” A value expressed by all participants was seeing their leaders follow recommendations to prevent burnout amongst clinicians, researchers, and staff.

A frequently cited example of poor leadership was when leaders showed a lack of internalized moral perspective. When this occurred, it seemed to suggest to participants that the leader was disingenuous. For example, one research faculty member complained that, “my perception of some leadership is that there’s hearing going on but not listening.”

#### Affinity group comparison-internalized moral perspective

Participants described in detail the importance of their leaders expressing internalized moral perspective, particularly as it related to the triplicate mission of clinical care, research and education. Across focus group discussions, the affinity groups agreed and appeared to feel the most strongly about their belief that for leaders to be trusted and appear genuine in their intentions, they had to demonstrate that their personal core values supported the mission of the organization (taking care of patients, conducting research, and education). No differences between affinity groups occurred for the leadership construct of internalized moral perspective.

### Self-awareness

The self-awareness construct in authentic leadership theory includes ways that leaders express how they make meaning of the world by evaluating their own values, motives, and emotions [24]. A self-aware leader seeks feedback from others about their leadership, and its impacts, and works to incorporate the feedback. This self-reflective process in turn informs the leader’s sense of self as a leader [10]. As discernable by the employees, a leader whose personal align with workplace decisions form the basis of this construct [26] and can “…foster positive self-development” in their employees [20].

Participants reported feeling better about work when their leaders were aware of and demonstrated their personal values or emotions through their actions as leaders. For instance, encouragement of wellbeing could occur if the leader expressed their own enjoyment of providing clinical care to their clinical faculty and staff, as highlighted by a clinical faculty member. “I think somebody who’s enthusiastic actually about being at the hospital, it’s kind of contagious and can kind of really push you as well to have kind of the same enthusiasm which kind of the way it starts from the top and from there it kind of spreads to every other person. So, I think always leading back to having a leader that actually is [genuinely] excited about work [would support employees feeling well at work].”

Participants described self-awareness from their leaders when they created and participated in organizational structures that would improve workplace interactions. As in this example, a research faculty member emphasized the importance of his leadership caring about being a member of a respectful work group and, in turn, facilitating staff interactions “in a collegial, respectful, helpful way, that really, I think, is the core of community in our research group.” Participants valued experiences of their leadership expressing self-awareness in the DOM.

### Affinity group comparison-self-awareness

The value to employees of seeing their leaders be self-aware was noted from study participants across the affinity groups. A difference was seen between the trainees affinity group compared to discussions with the other three affinity group participants. Trainees discussed personally valuing being a leader and they wanted to demonstrate leadership within their own group (to others in their cohort of fellows or residents). “As senior residents looking out for your junior residents and for your interns…if we want all of us to be friends and all of us to be helping each other out then we should be the ones performing that behavior and modeling it”, stated a senior resident. The other affinity groups participants agreed with valuing self-awareness in their leaders, yet they did not view themselves as leaders or as examples of leadership. They described wanting their division or hospital leaders to demonstrate their values of doctoring or management by enacting policies that matched these personal values. For instance, a clinical faculty member shared that she valued her leader reflecting on her personal value of providing good clinical care, and as a result, “maintaining a forty-minute visit in senior’s clinic…to have the time to actually provide good clinical care”.

In summary, study participants identified possible leadership attributes that could reduce burnout: giving others a voice in decision-making even when it challenges their own views, expressing and accepting emotional reactions to stressful situations, practicing transparency and sharing challenges affecting academic medicine today, and promoting collegiality and connectedness not in an artificial way, but because they personally value these types of interactions. Analyzing the results between affinity groups suggests the importance of leaders focusing on the local context of their employees’ work (clinical, educational, or research) to focus efforts to reduce burnout.

## Discussion

Burnout in healthcare is a very serious problem with important consequences. Therefore, it is vital to identify actionable items to not only reduce burnout but to ensure that employees thrive in their careers in academic medicine. Furthermore, the COVID pandemic has exacerbated the magnitude and severity of burnout at academic medical centers and among all healthcare professionals [27–30]. We found that the authentic leadership theory constructs provided a tool to analyze the experiences of leadership and understand factors related to burnout among academic clinicians, researchers, trainees, and administrative staff in an academic medical setting. 

There are several possible ways that authentic leadership reduces burnout and enhances wellness in healthcare providers. Organizational factors are associated with increased symptoms of burnout including inadequate social support, organizational politics, bullying in the workplace, employer unfairness, and leadership styles [31, 32]. An authentic leader focuses on the success of their team members. In a survey of over 300 nurses, authentic listening and communication by managers was positively associated with bedside nurse’s job and compassion satisfaction [33]. This survey demonstrated that leadership styles can shift the balance between compassion satisfaction and compassion fatigue thereby combatting the onset of emotional exhaustion and psychological stress. The results of this survey likely are applicable to other healthcare professionals in addition to nurses.

When a leader is engaged in listening to a team member’s needs and cognizant of their wellbeing, the leader may be more flexible and effective at shift schedules and determining daily responsibilities [34, 35]. In addition, a component of being an authentic leader, relational transparency, may help team members become more self-reliant and connected to the organization, thereby increasing their engagement in organizational activities and culture, and assuming increased control over their job. By enhancing this feeling of control, team members may reduce their emotional exhaustion and increase their feeling of personal accomplishment [18].

Some individuals are likely to gravitate inherently towards more of an authentic leadership style. However, like all personal traits, authentic leadership can be learned. There are several specific potential methods to enhance an authentic leadership style (Table 3). Unit based leaders can team with hospital administration to improve staff involvement in clinical decision making, such as participating in shift planning. Organizational culture can foster communication training based on emotional openness and transparency [18, 33]. Authentic leadership training could be embedded in professional development activities and made available to individuals in various leadership roles from charge nurses to small group instructors, to departmental chairs and hospital c-suite employees [18]. Healthcare leadership experts have suggested that leadership skills are critical at every level and across the entire range of healthcare providers [16]. Similarly, leadership training could occur earlier in training and be incorporated into medical, nursing, and graduate school curricula. These programs could be based upon action learning principles and focus on authentic leadership development by working through real problems, activities, and case studies. In addition, healthcare professional schools and hospitals could consider recruiting their leaders based on possessing an authentic leadership style [16].

**Table 3:** Potential solutions

1. Model shared decision making on a unit or divisional level	
2. Develop formal communication training to enhance openness and transparency	
3. Develop formal authentic leadership training for a variety of leadership positions	
4. Embed authentic leadership training into medical, nursing, and graduate school	
5. Recruitment of future leadership positions should access candidate’s leadership style and strive to hire individuals with an authentic leadership style.	

The limitations of this study include participants who were volunteers that selected these issues as a topic of interest. Although our goal was to conduct this study at as many sites as possible, some DOM members may have been excluded because of their location, given the broad geographic distribution of the DOM. We did not solicit the perspectives and experiences of DOM leaders. However, the experiences and perceptions of leaders would provide a valuable perspective and would complement the focus group findings of healthcare professionals’ experiences of leadership attributes in an academic medical center. The analyses conducted comparing results by affinity group are limited by an unequal number of focus groups conducted per affinity group and limitations stemming from the finite ability of qualitative research methods to compare results by subgroups. Nonetheless, the insights gained from comparing the affinity groups’ findings may prove insightful for medical leadership targeting their efforts to address burnout specifically to employee types or roles within the healthcare institution. Finally, we focused on an authentic leadership style. The most effective leaders utilize multiple leadership styles depending on the situational context.

Further work is needed to more definitively determine if identifying and selecting leaders who have attributes aligned with an authentic leadership style may help combat burnout in spite of mounting pressure including a reduction in national research funding, increased demands of clinical revenue, and increased administrative burden to all. Importantly, if found to change these outcomes, an authentic leadership style includes skills and attributes that can be trained and reinforced in leaders in academic medicine--making this an actionable finding for a widespread problem.

## Conclusion

Our qualitative exploration of academic clinicians, researchers, trainees, and administrative staff adds to the growing literature that leadership styles can be an important mediator of burnout in academic medical centers. We identified that an authentic leadership style that promoted relationship transparency, openness, and support was desired by all of the affinity groups. Adoption of an authentic leadership style at various levels in academic medical centers could help mitigate the growing burnout crisis in healthcare. Future studies will be needed to determine the best methods to teach authentic leadership styles to emerging healthcare leaders. Similarly, future research is necessary to determine the most effective method to recruit individuals who utilize an authentic leadership style in order to enhance job satisfaction and improve retention of our healthcare workforce.

## Supplementary Information

**Additional file 1.**

## Data Availability

The datasets used and/or analyzed during the current study are available from the corresponding author on reasonable request.
