# Supplementary material for: Burnout and the role of authentic leadership in academic medicine
Source: BMC Health Serv Res. 2022 May 11;22:627. doi: 10.1186/s12913-022-08034-x (PMC9092784; doi:10.1186/s12913-022-08034-x)
Supplement: Supplementary file 1 — Additional file 1. [file 12913_2022_8034_MOESM1_ESM.docx]

Understanding the factors that contribute to the development of a culture of wellness in the department of medicine

n = 12 focus groups

**IRB** Protocol #18-1775

FINAL version 11/12/18

Eligibility Criteria

- Employed by the Department of Medicine
- Full time employees
- Member of one of the defined affinity groups
- Completes/delivers signed consent form/pre focus group survey to focus group

Exclusion Criteria

- Served as a Member of the Wellness Initiative Committee

# I. Introductory Remarks/Welcome

Hi and welcome! Thank you for taking time out of your busy schedules for this focus group today. My name is <<MODERATOR>> and I will be the facilitator for this session. I am an “outside” consultant who was hired to facilitate twelve focus groups for the Department of Medicine’s Wellness Committee team. I am not connected with the Department of Medicine other than for this consulting project. With me is <<NOTETAKER>>, a member of this team, who will serve as note taker.

My role is to ask questions and watch the time so that we can do as much as we can during this 45 minute session. <<NOTETAKER>> will be taking notes as back up to the tape recording.

Hopefully you have taken the chance to go to the link provided in your invitation, read the consent form and fill out the pre-focus group survey. If you haven’t done this yet, please take the time to complete it afterwards.

There will be a final report that comes out of the twelve focus groups that we are conducting. Please know that your name will not be associated with the responses that we note in the aggregate report.

In order to get all of your great input, and because <<NOTETAKER>> will not be able to write as fast as you talk, we would like to audio-record this session. Information you offer in this focus group is intended for the sole purpose of the project and will remain confidential. That is, people not associated with the project will not hear the audio recording or know what you shared and any materials associated with this focus group discussion will not have your name or identifiable characteristics. Is everyone okay with this? Do you have any questions?

By show of hands, please indicate your consent to participate in this focus group and be audio-recorded.

We consider all of you around the table today as the experts who can help with the goal of mitigation of Burnout Syndrome in an effective and sensitive manner. As <<AFFINITY GROUP name>> and employees of the Department of Medicine, you work in the environment that the team wants to address as part of this effort. We want to listen and learn from you and other affinity groups to get your reaction to some solutions proposed by your colleagues during a recent retreat and to get your thoughts on how to engender a culture of wellness on the four hospital campuses (Denver Health, National Jewish, University, and VA hospitals).

There are no right or wrong answers in this focus group- something that one person says or believes might be different from what another person says or believes. We want you to feel free to share any comments that you have- both positive and negative. Most importantly, we’d like to hear your thoughts and opinions.

We’re going to talk for about 45 minutes and we will definitely be done by <<TIME>> as was promised in your initial agreement for being here.

The way this focus group works is that I’m going to ask you some questions and make sure that everyone has an opportunity to respond. You can choose not to answer, but it helps if everyone participates. I am about to turn on the audio recording for this session but does anyone have any questions before we start?

**II. Focus Group Questions (start by <<TIME>> 45 minutes remaining for discussion)**

**Q1.** (Icebreaker) Let’s get started by going around the room and if you could each tell us: (1) your first name; (2) division; and (3) which hospital where you have primary appointment?

- For <<TRAINEES>> and (4) please note if you are a Fellow or Resident
- For <<CLINICAL FACULTY>> and (4) if you have a primary IN-PATIENT or OUT-PATIENT focus

NOTETAKER please NOTE this on the table diagram for analysis later.

Great- welcome and thanks for coming. I’m going to ask questions about the current state of what we are calling a “Culture of Wellness” in the Department of Medicine including some strategies to improve this culture within our discussion.

**Culture of Wellness**

The first topic for discussion is in regards to a goal or desire for a “Culture of Wellness” for the Anschutz (and related hospitals) campus. By “Culture of Wellness” we mean: the fostering of professional community spirit and well being in the Department of Medicine with a lessening or absence of Burnout Syndrome.

**Q2**. What are some competencies, characteristics or behaviors that leaders/supervisors should have in order to drive a culture of wellness on the (4 hospital) campus?

**Q3.** Please identify some things that you or others in your affinity group currently do or could do

to help foster community and well-being in the DOM. (probe: What are some competencies, characteristics or behaviors that you or your peers in this affinity group of <<trainees, clinical faculty, research faculty, staff>> currently utilize or could utilize to help create a culture of wellness?)

**Enhancing Human Connection within the Department**

For this next part, think about your current experience with colleagues – specifically about how you connect on a human level in the name of building community within the Department of Medicine.

**Q4**. In what ways do you currently experience connection with your colleagues? (probe: what activities, settings, help with an experience of human connection with your <<affinity group>> colleagues?)

**Q5.** If we generally agree that connection with colleagues is important, how do you imagine increasing opportunities for interpersonal connection and mutual support within the DOM?

(probe: what WAYS, METHODS, TANGIBLE ACTIONS or INITIATIVES can be done that could make a difference on these campuses?)

**Q6.** In what way do you imagine yourself participating in initiatives aimed at enhancing connection?” (probe: we are not asking for a specific commitment from you today, but where can you see YOURSELF or PEOPLE IN YOUR AFFINITY GROUP playing a role in any of the methods/initiatives just described?)

Now that we have had a chance to talk about what a culture of wellness can look like, let’s take a look at what might be getting in the way of a true “Culture of Wellness” on campus.

**Q7.**What do you see as potential barriers for a Culture of Wellness to exist on this campus and with the related hospitals mentioned?

**Q8.** One barrier mentioned in a recent retreat about this topic was an expressed feeling of separation in vision between the DOM and the hospitals where members of the DOM work. In what way(s) do you feel the hospital where you work and the DOM have differences in core values, mission and vision?

**Q9.** In what way(s) do you feel that those differences impact the wellness and thriving of DOM members?

**III. Closing <<with 5 minutes left>**

**Q10.** We are at the final question and we want to be sure we captured everybody’s thoughts today on this issue of mitigating Burnout Syndrome for DOM employees through a Culture of Wellness lens. Is there anything else that you didn’t get to share that will help the Wellness Committee team as it moves forward with implementation of solutions in a sensitive and efficient manner?

*Martha turns to <<NOTETAKER>> to see if there is anything to further probe or if she wants to clarify anything that has been said.*

**Other Questions/Closing Remarks**

THANK YOU so much for your time and great comments!

IV. After the Focus Group

Breakdown of focus group equipment.

Quick debrief (allow up to 20 minutes for this) with <<NOTETAKER>>, using the Focus Group Debrief Summary forms. Martha will facilitate this process and write up these notes.

<<NOTETAKER>> hands over diagram of table and fieldnotes/emails notes (if took notes on a laptop) to Martha. Martha will scan/submit these notes and the digital audiotape to the transcriptionist within 24 hours of focus group session.
